# Occurrence of Presumptive Methicillin-Resistant *Staphylococcus aureus* and Vancomycin-Resistant Enterococci in Retail Fish and Seafood Products: A Culture-Based Screening Study

**DOI:** 10.3390/foods15142519

**Published:** 2026-07-16

**Authors:** Anita Kukułowicz

**Affiliations:** Department of Quality Management, Faculty of Management and Quality Sciences, Gdynia Maritime University, 81–87 Morska St., 81-225 Gdynia, Poland; a.kukulowicz@wznj.umg.edu.pl

**Keywords:** antimicrobial resistance, seafood contamination, retail food safety, foodborne bacteria, hygienic handling, aquatic food products

## Abstract

Background: Fish and seafood products may act as vehicles for the dissemination of antimicrobial-resistant bacteria through the food chain. Particular concern is associated with methicillin-resistant *Staphylococcus aureus* (MRSA) and vancomycin-resistant enterococci (VRE). This study evaluated the occurrence of *S. aureus*, *Enterococcus* spp., and presumptive MRSA and VRE in retail fish and seafood products using culture-based screening methods. Methods: A total of 99 fish and seafood samples purchased from retail outlets in northern Poland were analyzed using selective microbiological culture methods. Presumptive MRSA and VRE were screened on chromogenic selective media. Differences between product categories were assessed using non-parametric statistical tests and chi-square analysis. Results: *S. aureus* was detected in 80.8% of samples, with significantly higher contamination levels in seafood than in fish (*p* = 0.002). Presumptive MRSA isolates were detected in 27.3% of samples. *Enterococcus* spp. were found in 49.5% of samples, with significantly higher prevalence and counts in unpackaged products (*p* < 0.05). Presumptive VRE isolates occurred in 17.2% of samples, while simultaneous occurrence of presumptive MRSA and VRE was observed in 9.1% of samples. Conclusions: Retail fish and seafood products may constitute a reservoir of presumptive antimicrobial-resistant bacteria. The higher contamination of unpackaged products highlights the importance of hygienic handling practices. Molecular confirmation and antimicrobial susceptibility testing are needed to further assess the public health significance of these microorganisms.

## 1. Introduction

Fish and seafood constitute an important component of the human diet and represent a significant sector of global food production, including both capture fisheries and aquaculture. According to the European Market Observatory for Fisheries and Aquaculture Products [1], the apparent consumption of fishery and aquaculture products in the European Union reached approximately 22.9 kg per capita in 2023. In Poland, fish consumption remains lower than the EU average; in 2023 the average consumption amounted to 13.7 kg per capita [2]. Despite this lower level of consumption, fish and seafood products are widely available in retail distribution, highlighting the importance of monitoring their microbiological quality and safety. Due to the high water activity and biochemical composition of fish tissues, these products are particularly susceptible to microbial contamination and spoilage. The microbiological profile of fish is strongly influenced by microorganisms present in the aquatic environment, as well as by factors such as species, feeding habits, season, water temperature, and handling conditions during capture and processing [3]. In living fish, microorganisms are mainly located on external surfaces such as the skin and gills and in the intestinal tract, whereas the muscle tissue of healthy fish is typically sterile. After the death of the fish, however, bacterial proliferation may occur and microorganisms can invade muscle tissue, contributing to spoilage and potential health risks for consumers [3]. Contamination of fish and seafood may occur both in the natural aquatic environment and at subsequent stages of the food chain, including harvesting, processing, transport, storage, and retail distribution. Retail outlets represent the final stage of this chain; therefore, the microbiological quality of products available for sale reflects both environmental conditions and hygienic practices during handling and processing [4,5,6].

The use of antibiotics in aquaculture has been associated with the emergence and dissemination of antimicrobial-resistant bacteria in aquatic environments. Previous studies indicate that antimicrobial agents applied in fish farming may contribute to the development of resistance in several bacterial species, including *Enterococcus faecalis* [7]. Furthermore, antibiotic residues and resistant bacteria present in aquaculture systems may facilitate the spread of antimicrobial resistance genes within aquatic ecosystems [8]. Consequently, fish and seafood may act as vehicles for the transmission of multidrug-resistant (MDR) bacteria into the food chain, posing a risk to consumer health [9].

Among bacteria associated with contamination of fish and seafood products, *Staphylococcus aureus* is considered one of the most important foodborne pathogens [10]. This microorganism commonly colonizes human skin and mucous membranes and may be transferred to food during handling and processing [11]. The presence of *S. aureus* in fish products is therefore often considered an indicator of secondary contamination associated with inadequate hygiene during processing or retail handling [4,12,13]. Of particular concern is methicillin-resistant *Staphylococcus aureus* (MRSA), which has increasingly been detected not only in clinical settings but also in retail foods [14,15], including fish and seafood products, raising concerns regarding the dissemination of antimicrobial resistance through the food chain. Previous studies have reported the occurrence of MRSA in raw meat and other food products, suggesting that food may act as a potential source of antibiotic-resistant bacteria for consumers [16].

Enterococci constitute another group of bacteria frequently detected in foods and environmental samples. These microorganisms are widely distributed in soil, water, plants, and in the gastrointestinal tract of humans and animals. In food microbiology, *Enterococcus* spp. are often considered indicators of hygienic conditions during food processing and handling [6,17,18]. Moreover, enterococci are recognized as opportunistic pathogens associated with hospital-acquired infections and are characterized by a high capacity for environmental persistence and acquisition of antimicrobial resistance determinants. In particular, vancomycin-resistant enterococci (VRE) represent an important epidemiological concern because of their ability to acquire, maintain, and transfer antimicrobial resistance determinants to other Gram-positive bacteria. Recent studies have demonstrated the occurrence of enterococci exhibiting virulence factors and resistance determinants, including resistance to vancomycin and linezolid, in fish and crustaceans [16,19,20].

An additional concern is the potential transfer of resistance genes between bacterial species. The vanA gene, responsible for vancomycin resistance, has been identified in both enterococci and vancomycin-resistant *Staphylococcus aureus* (VRSA). Genomic analyses have shown that the vanA locus may become integrated into the chromosome of *S. aureus*, suggesting the potential of genetic exchange between enterococci and staphylococci [16,21]. Such mechanisms highlight the role of food products as potential reservoirs for antimicrobial resistance genes.

Despite the growing number of studies on antimicrobial-resistant bacteria in foods of animal origin, data regarding the occurrence of *S. aureus*, including methicillin-resistant strains (MRSA), and vancomycin-resistant enterococci in retail fish and seafood remain limited. Monitoring these microorganisms in seafood products is important for assessing potential risks related to the dissemination of antimicrobial resistance within the food chain and potential consumer exposure to resistant bacteria through retail food products.

Therefore, the aim of this study was to evaluate the occurrence of *S. aureus*, *Enterococcus* spp., and presumptive MRSA and VRE in retail fish and seafood products using culture-based screening methods, and to assess potential associations with product type, preservation method, and packaging conditions.

## 2. Materials and Methods

### 2.1. Material

The study was conducted on fish and seafood products, including fish, crustaceans, and mollusks, purchased from retail outlets located in Gdynia, northern Poland. Samples were collected between February and June 2025 using a convenience sampling approach based on product availability at the time of purchase. This sampling approach may limit the representativeness of the analysed material and should be considered when interpreting the results. A total of 99 samples were analyzed. The samples were obtained from different types of retail outlets, including a large supermarket chain, a local fish market, and a specialised fish shop. The analysed material included various fish species, as well as seafood products such as shrimp, mussels, squid, and mixed seafood products. For the purpose of the study, the samples were classified into two main categories according to product type: fish and seafood. Additionally, the samples were categorised according to preservation status and packaging conditions. Based on preservation status, the products were classified as frozen or fresh. According to packaging conditions at the point of sale, the samples were classified as packaged or unpackaged. The distribution of the analysed samples is presented in Table 1.

The study did not involve live animals or any experimental procedures performed on animals. All analysed samples consisted of fish and seafood products purchased from retail outlets as food products intended for human consumption. Consequently, ethical approval and ARRIVE reporting guidelines were not applicable to this study.

The purchased products were transported to the microbiological laboratory in insulated cooling containers with ice packs to maintain refrigerated conditions and preserve the cold chain during transport. The transportation time was approximately 45 min. All samples were analysed immediately upon arrival at the laboratory on the day of purchase.

### 2.2. Microbiological Analysis

Twenty-gram portions of each sample were collected aseptically in a laminar air-flow cabinet and homogenized with 180 mL of sterile physiological saline using a Stomacher Lab-Blender 400 (Seward, Worthing, UK), resulting in an initial 0.1 dilution. Subsequent decimal dilutions were prepared as required.

### 2.3. Enumeration of Staphylococcus aureus

Enumeration of *S. aureus* was performed according to ISO 6888-1:2001 [22]. From appropriate dilutions, 1 mL aliquots were plated using the pour-plate technique on Baird-Parker agar supplemented with RPF (bioMérieux, Warsaw, Poland). The plates were incubated at 37 °C for 24–48 h. Typical black colonies surrounded by a clear halo on Baird-Parker agar were considered presumptive *S. aureus* and were confirmed by catalase and coagulase tests [22,23,24]. The limit of quantification of the method was 10 CFU/g.

### 2.4. Screening of Presumptive Methicillin-Resistant Staphylococcus aureus (MRSA)

For the screening of presumptive methicillin-resistant *Staphylococcus aureus* (MRSA), suspensions obtained from homogenized samples were subjected to initial incubation in Giolitti-Cantoni broth (Merck KGaA, Darmstadt, Germany). The medium was overlaid with approximately 2 cm of molten sterile agar and incubated at 37 °C for 24 h. Samples showing blackening of the medium were subsequently surface-plated onto a CHROMagar MRSA base (Graso Biotech, Gdańsk, Poland) by spreading 1 mL of the enrichment culture over the agar surface. Plates were incubated for an additional 24 h at 37 °C. According to the manufacturer’s instructions, pink colonies were interpreted as presumptive MRSA isolates [25]. The use of selective chromogenic medium alone does not provide definitive confirmation of methicillin resistance.

### 2.5. Detection and Enumeration of Enterococcus spp.

For the detection of *Enterococcus* spp., homogenized samples were inoculated into selective azide dextrose broth (Merck, Darmstadt, Germany) and incubated at 35 °C for 24–48 h. The development of turbidity was considered indicative of enterococcal growth. Positive cultures were subsequently transferred by inoculating 1 mL of the enrichment culture into Bromocresol Purple Azide Broth (Merck) and incubated at 36 ± 1 °C for up to 48 h. The presence of turbidity accompanied by a change in the color of the medium from purple to yellow confirmed the presence of enterococci. For isolation and enumeration, appropriate decimal dilutions prepared from the homogenized samples were inoculated (1 mL) onto the surface of Slanetz–Bartley agar (Merck, Darmstadt, Germany) and incubated at 37 °C for 48 h. Typical dark red or maroon colonies were presumptively identified as *Enterococcus* spp. Presumptive isolates were further confirmed by Gram staining, catalase testing, assessment of gas production from mannitol, and evaluation of growth in the presence of 6.5% NaCl [26,27,28]. Identification based on these phenotypic characteristics does not provide definitive species identification and should therefore be regarded as presumptive. The limit of quantification of the method was 10 CFU/g.

### 2.6. Screening of Presumptive Vancomycin-Resistant Enterococci (VRE)

For the screening of presumptive vancomycin-resistant enterococci (VRE), suspensions obtained from homogenized samples were initially enriched in selective sodium azide dextrose broth (Merck, Darmstadt, Germany) at 35 °C for 24–48 h. Cultures showing turbidity were subsequently streaked onto CHROMID^®^ VRE agar (bioMérieux, Warsaw, Poland) and incubated at 37 °C for 24–48 h. According to the manufacturer’s instructions, bluish-green colonies were interpreted as presumptive *Enterococcus faecalis*, whereas violet colonies were interpreted as presumptive *Enterococcus faecium* [29,30]. As the identification was based on selective chromogenic medium, the isolates should be regarded as presumptive, and further confirmatory testing would be required to verify vancomycin resistance.

The numbers of *S. aureus*, presumptive MRSA, *Enterococcus* spp., and presumptive VRE were calculated in accordance with PN-EN ISO 7218:2008 [31] and expressed as colony-forming units per gram (CFU/g). The detection limit of the enumeration method was 10 CFU/g.

### 2.7. Statistical Analysis

Due to the non-normal distribution of microbiological counts and the high proportion of samples below the detection limit, the data were analysed using non-parametric statistical methods. Values below the limit of quantification (<10 CFU/g) were recorded as 0 CFU/g for statistical analyses and are presented as <10 CFU/g in the manuscript to reflect the analytical limit of quantification. Bacterial counts were expressed as medians and interquartile ranges (Q1–Q3). Comparisons between two independent groups (fish vs. seafood, frozen vs. fresh products, and packaged vs. unpackaged products) were performed using the Mann–Whitney U test. When more than two groups were compared (supermarket chain, fish market, and specialised fish shop), the Kruskal–Wallis test was applied. The prevalence of the analysed microorganisms was evaluated using the chi-square (χ^2^) test. Effect sizes were calculated using the r statistic for Mann–Whitney U tests and Cramér’s V for chi-square analyses. Due to the exploratory nature of the study and the limited number of predefined comparisons, formal correction for multiple testing was not applied. Therefore, *p*-values, particularly those close to the significance threshold, should be interpreted with caution. The level of statistical significance was set at *p* < 0.05. Statistical analyses were performed using Statistica Version 13 (StatSoft, Inc., Tulsa, OK, USA, 2013) [32].

## 3. Results

A total of 99 samples were analysed, including 58 fish samples and 41 seafood samples. The analysed material consisted of 42 frozen and 57 fresh products, of which 54 were packaged and 45 were sold unpackaged (Table 1). Detailed microbiological results for each analysed sample are presented in Supplementary Table S1.

### 3.1. Occurrence of Enterococcus spp. and Presumptive VRE

*Enterococcus* spp. were detected in 49 samples (49.5%), including 28 fish samples (48.3%) and 21 seafood samples (51.2%) (Table 2). Presumptive VRE isolates were detected in 17 samples (17.2%), including 8 fish samples (13.8%) and 9 seafood samples (22.0%).

Median counts of *Enterococcus* spp. were <10 CFU/g (Q1–Q3: <10–40) in fish and 10 CFU/g (<10–150) in seafood samples, with no statistically significant differences between the analysed groups (*p* = 0.219) (Table 3). Median counts of presumptive VRE remained below the detection limit in both product categories.

With respect to preservation status, *Enterococcus* spp. were detected in 18 frozen samples (42.9%) and 31 fresh samples (54.4%). Median bacterial counts were <10 CFU/g (<10–40) in frozen products and 10 CFU/g (<10–100) in fresh products; however, the differences were not statistically significant (*p* = 0.237) (Table 4). Presumptive VRE isolates were detected in 5 frozen samples (11.9%) and 12 fresh samples (21.1%), with no statistically significant differences between the analysed groups (*p* > 0.05).

Packaging conditions significantly affected the occurrence of enterococci. *Enterococcus* spp. were detected in 21 packaged samples (38.9%) and 28 unpackaged samples (62.2%). Median counts were <10 CFU/g (<10–37.5) in packaged products and 30 CFU/g (<10–110) in unpackaged products, with significantly higher levels observed in unpackaged samples (*p* = 0.023) (Table 5). Presumptive VRE isolates were detected in 7 packaged samples (13.0%) and 10 unpackaged samples (22.2%), although the differences were not statistically significant (*p* > 0.05).

The highest level of *Enterococcus* spp. in fish was detected in fresh unpackaged fish purchased at a fish market, reaching 1.7 × 10^3^ CFU/g, with simultaneous detection of presumptive VRE at 1.6 × 10^2^ CFU/g. In seafood samples, the highest level of *Enterococcus* spp. was detected in fresh unpackaged seafood purchased from a supermarket, reaching 7.2 × 10^3^ CFU/g, with simultaneous detection of presumptive VRE at 80 CFU/g. Presumptive vancomycin-resistant enterococci (VRE) were detected in 17 samples (17.2%), including 8 fish samples (13.8%) and 9 seafood samples (22.0%) (Table 2). Median counts in both product groups remained below the detection limit, and no statistically significant differences were observed between fish and seafood samples (*p* = 0.302) (Table 3). Presumptive VRE isolates were detected in 5 frozen samples (11.9%) and 12 fresh samples (21.1%), as well as in 7 packaged samples (13.0%) and 10 unpackaged samples (22.2%), with no statistically significant differences between the analysed groups (*p* > 0.05).

### 3.2. Occurrence of Staphylococcus aureus and Presumptive MRSA

*S. aureus* was the most frequently detected microorganism and was identified in 80 samples (80.8%) (Table 2). The bacterium was detected in 43 fish samples (74.1%) and 37 seafood samples (90.2%). Median counts were 20 CFU/g (2.5–25) in fish and 90 CFU/g (20–210) in seafood samples, with significantly higher contamination levels observed in seafood products (*p* = 0.002) (Table 3). With respect to preservation status, *S. aureus* was detected in 36 frozen samples (85.7%) and 44 fresh samples (77.2%). Median counts were 30 CFU/g (20–180) in frozen products and 30 CFU/g (10–120) in fresh products, with no statistically significant differences between the analysed groups (*p* = 0.216) (Table 4).

Higher contamination levels were also observed in unpackaged products. *S. aureus* was detected in 39 packaged samples (72.2%) and 41 unpackaged samples (91.1%), with median counts of 20 CFU/g (<10–142.5) and 30 CFU/g (20–180), respectively. However, the observed differences were not statistically significant (*p* = 0.052) (Table 5).

The highest level of *S. aureus* in fish was detected in fresh packaged fish purchased from a supermarket, reaching 3.6 × 10^2^ CFU/g, with simultaneous detection of presumptive MRSA at 20 CFU/g. In seafood samples, the highest contamination level was observed in fresh unpackaged seafood purchased from a supermarket, reaching 2.4 × 10^3^ CFU/g, where presumptive MRSA reached 1.0 × 10^2^ CFU/g.

Presumptive MRSA isolates were detected in 27 samples (27.3%), including 13 fish samples (22.4%) and 14 seafood samples (34.1%) (Table 2). Median counts remained below the detection limit in both product categories, and no statistically significant differences were observed between fish and seafood samples (*p* = 0.220) (Table 3). Presumptive MRSA isolates were detected in 11 frozen samples (26.2%) and 16 fresh samples (28.1%), as well as in 13 packaged samples (24.1%) and 14 unpackaged samples (31.1%), with no statistically significant differences between the analysed groups (*p* > 0.05). The highest presumptive MRSA level in fish was observed in fresh unpackaged fish purchased at a fish market, reaching 90 CFU/g, whereas in seafood samples the highest value (1.1 × 10^2^ CFU/g) was recorded in frozen packaged seafood obtained from a fish market.

### 3.3. Co-Occurrence of Presumptive MRSA and VRE

Simultaneous occurrence of presumptive MRSA and presumptive VRE was detected in 9 samples (9.1%), including 5 fish and 4 seafood samples. Most of these cases were associated with fresh products (8 samples), whereas only one occurred in a frozen product. A statistically significant association between the occurrence of presumptive MRSA and presumptive VRE was observed (*p* = 0.021). A chi-square (χ^2^) test was performed to evaluate differences in the prevalence of the analysed microorganisms between product categories, preservation status, and packaging conditions. No statistically significant differences in the prevalence of *Enterococcus* spp., presumptive VRE, *S. aureus*, or presumptive MRSA were observed between fish and seafood samples or between frozen and fresh products (*p* > 0.05). In contrast, unpackaged products showed a significantly higher prevalence of *Enterococcus* spp. (*p* = 0.035) and *S. aureus* (*p* = 0.034) compared with packaged products.

No statistically significant differences in bacterial counts were observed between retail outlet types (supermarket chain, fish market, and specialised fish shop) for any of the analysed microorganisms (*p* > 0.05).

## 4. Discussion

*Staphylococcus aureus* is one of the most important foodborne pathogens associated with fish and seafood products and is commonly regarded as an indicator of secondary contamination resulting from inadequate hygienic practices during processing, handling, and retail distribution [4,6,33]. In the present study, *S. aureus* was detected in 80.8% of the analyzed samples, with significantly higher contamination levels observed in seafood compared with fish (*p* = 0.002; Table 3). Similar prevalence values were reported in Japan, where *Staphylococcus* spp. were detected in 87% of sashimi products [34]. In Poland, Kukułowicz et al. [33] reported a lower overall prevalence of *S. aureus* in fish and seafood products (59%), with bacterial counts ranging from below the detection limit to 3.1 × 10^2^ CFU/g, which was lower than the maximum level observed in the present study (2.7 × 10^3^ CFU/g). The prevalence of *S. aureus* reported in the literature varies considerably depending on product type, geographical region, hygienic conditions, and retail practices. Odetokun et al. [35], in a systematic review concerning foods of animal origin, reported prevalence values ranging from 1.3% to 72.5%. Lower prevalence levels in fishery products were also reported in Nigeria (16.5%) [36], north-western Spain (25.2%) [37], Iran (34%) [38], and Egypt (39.4%) [39]. The relatively high prevalence observed in the present study may suggest substantial environmental exposure and secondary contamination occurring during processing, retail display, or handling of seafood products. The significantly higher contamination levels observed in seafood compared with fish may additionally reflect differences in product handling, processing intensity, surface characteristics, or the greater diversity of seafood products included in the analysed material. No statistically significant differences in *S. aureus* prevalence were observed between fresh and frozen products (*p* = 0.216). Since the analysed fresh and frozen products did not originate from the same production batches, these findings should not be interpreted as evidence that freezing has no effect on bacterial survival. Rather, the comparable contamination levels observed in both product categories suggest that contamination may occur at multiple stages of the seafood supply chain regardless of preservation status. Similar inconsistencies have been reported previously. Sivaraman et al. [40] observed a twofold higher prevalence of *S. aureus* in fresh fish than in frozen products, whereas Dallal et al. [41] reported the opposite trend. Comparable prevalence values in fresh and frozen fishery products were also reported by Kukułowicz et al. [33]. Differences in the prevalence of *S. aureus* observed across studies may result from variations in hygienic practices, storage conditions, and processing and distribution procedures, as contamination may occur at multiple stages of the food chain, including fish processing facilities and retail environments [13,35,42]. In the present study, unpackaged products exhibited a significantly higher prevalence of *S. aureus* than packaged products (*p* = 0.034), whereas differences in bacterial counts approached but did not reach statistical significance (*p* = 0.052). This finding suggests that direct exposure to personnel, equipment, and the surrounding retail environment may contribute to secondary contamination. Food handlers may represent an important source of contamination in seafood processing and retail environments. Simon and Sanjeev [12] reported that 62% of fish-processing workers carried *S. aureus*, while enterotoxigenic strains were also identified among isolates recovered from workers, indicating the potential transfer of these bacteria to seafood products during processing and handling.

Presumptive methicillin-resistant *Staphylococcus aureus* (MRSA) isolates were detected in 27.3% of analysed samples. Although molecular confirmation of methicillin resistance was not performed, the results indicate the occurrence of presumptive antibiotic-resistant staphylococci in retail fish and seafood products. Similar observations were reported by Arfatahery et al. [38], who detected the mecA gene in 23.8% of *S. aureus* isolates, while 84.9% of the strains exhibited resistance to at least one antimicrobial agent. Hammad et al. [34] detected *Staphylococcus* spp. in 92% of surveyed stores selling sashimi products and reported MRSA in 5% of analysed samples. Similarly, Mohammed et al. [39] found that 46.2% of *S. aureus* isolates obtained from oysters sold in Egypt were identified as MRSA and exhibited multidrug resistance. Lower prevalence values were reported in Nigeria, where MRSA accounted for 2.3% of analysed samples and 14% of *S. aureus* isolates [36]. Derke et al. [13] identified MRSA in 5.21% of *S. aureus* isolates, whereas Sivaraman et al. [40] detected MRSA in only 3% of analysed seafood samples. The occurrence of antibiotic-resistant bacteria in fishery products has also been documented in Central Europe, including the Slovak retail market [43]. Fish and seafood are not considered natural reservoirs of MRSA; therefore, the presence of presumptive MRSA isolates in retail products is most likely associated with environmental contamination or secondary contamination occurring during processing, transport, storage, and retail handling [13]. Numerous studies have demonstrated that *S. aureus* isolates obtained from foods of animal origin frequently exhibit multidrug resistance, particularly to β-lactam antibiotics, highlighting the increasing importance of this pathogen in the context of food safety and public health [35]. The detection of presumptive MRSA in retail seafood products therefore indicates the potential dissemination of antimicrobial-resistant bacteria through the food chain and warrants further investigation using molecular confirmation and antimicrobial susceptibility testing.

In the present study, *Enterococcus* spp. were detected in 49.5% of analysed samples, with significantly higher prevalence and bacterial counts observed in unpackaged products (*p* = 0.023). Enterococci are commonly regarded as indicators of hygienic conditions during food processing and handling [17,44]. The higher contamination levels observed in unpackaged products suggest greater exposure to environmental contamination and more intensive handling during retail sale. Similar trends were previously reported by Kukułowicz et al. [44] in studies conducted in the same geographical region.

The prevalence of *Enterococcus* spp. observed in the present study was comparable between fish and seafood products (48.3% and 51.2%, respectively), and no statistically significant differences were detected between these product categories. Similarly, no significant differences were observed for presumptive MRSA and presumptive VRE. These findings may suggest that contamination is driven more by post-harvest handling, retail practices, and environmental exposure than by the biological characteristics of the analysed product categories. Comparable or higher prevalence values for enterococci have been reported in Poland (58.1) [44], Turkey (60%) [18], and Bangladesh (66%) [9], whereas lower values were observed in Turkey (21%) [6], India (28.7) [45], and Saudi Arabia (32.7) [20]. Krahulcová et al. [43] detected enterococci in 74% of sushi samples, with bacterial counts reaching 1.6 × 10^4^ CFU/g, which was comparable to the maximum levels observed in the present study. Beyond their role as indicators of hygienic conditions, enterococci are increasingly recognized as opportunistic pathogens associated with hospital-acquired infections and antimicrobial resistance dissemination [17]. Their ability to survive adverse environmental conditions, persist on food-processing surfaces, and acquire resistance determinants makes them particularly relevant from a food safety perspective. The presence of enterococci in seafood products may result both from their natural occurrence in aquatic environments and from secondary contamination during processing and retail distribution [17,44]. In addition, contamination may occur during evisceration, when microorganisms originating from the gastrointestinal tract are transferred to fish muscle tissue [43].

Presumptive vancomycin-resistant enterococci (VRE) were detected in 17.2% of analysed samples. Although molecular confirmation of resistance determinants was not performed, this finding suggests the occurrence of presumptive vancomycin-resistant enterococci in seafood products available at the retail level. Previous studies have demonstrated the presence of vancomycin-resistant enterococci and vanA-positive strains in fishery products [20,45]. Renukuttan et al. [45] identified the vanA gene in *Enterococcus faecium* isolates recovered from seafood products, whereas Abdel-Raheem et al. [20] reported the occurrence of enterococci resistant to both vancomycin and linezolid in fish and crustaceans. Higher levels of vancomycin resistance among enterococci were reported by Igbinosa and Beshiru [5] and Kukułowicz et al. [44]. Furthermore, Rana et al. [9] demonstrated the presence of multiple resistance determinants and virulence-associated genes in *Enterococcus faecalis* isolated from cultured and wild fish. Together, these findings suggest that fishery products may act as potential reservoirs of antimicrobial-resistant enterococci and may contribute to the dissemination of resistance determinants within aquatic and food-associated ecosystems.

The simultaneous occurrence of presumptive MRSA and presumptive VRE in 9.1% of samples may have important epidemiological implications. Enterococci are recognized reservoirs of antimicrobial resistance genes and have the capacity to exchange genetic material with other Gram-positive bacteria [16]. Previous genomic studies have demonstrated that vancomycin resistance determinants, particularly the vanA gene cluster, may occur in both enterococci and vancomycin-resistant *Staphylococcus aureus* (VRSA) strains [21,46]. Although the present study did not include molecular characterization of isolates, the co-occurrence of presumptive resistant bacteria within the same food products may indicate conditions favourable for the persistence and dissemination of antimicrobial resistance within seafood-associated microbial communities.

This study has several limitations. Samples were collected from a limited number of retail outlets within a single geographic region using a convenience sampling approach. Furthermore, presumptive MRSA and presumptive VRE were identified using culture-based screening methods without molecular confirmation or antimicrobial susceptibility testing. In addition, the performance of the selective chromogenic media was not verified using reference quality control strains, which represents a further methodological limitation of this screening study. Consequently, the findings should be interpreted as screening data rather than confirmation of resistant strains. Future studies incorporating molecular characterization and resistance profiling are warranted.

## 5. Conclusions

Retail fish and seafood products were frequently contaminated with *Staphylococcus aureus* and *Enterococcus* spp., while presumptive MRSA and presumptive VRE were also detected using culture-based screening methods. Higher contamination levels observed in unpackaged products indicate the importance of hygienic handling practices during retail sale. Although the presence of presumptive antimicrobial-resistant bacteria was observed, these findings should be interpreted with caution because no molecular confirmation or antimicrobial susceptibility testing was performed. Given the exploratory nature of the study and the relatively limited sample size, further investigations involving larger datasets and confirmatory analyses are needed to better characterize the occurrence and epidemiological significance of antimicrobial-resistant bacteria in retail fish and seafood products.

## Figures and Tables

**Table 1:** Characteristics of analysed samples.

Type of Samples		
Product type		
Fish		n ^1^ = 58
Seafood		n = 41
	Preservation status	
Frozen (F)		n = 42
Fresh (R)		n = 57
	Packaging condition	
Packaged (P)		n = 54
Unpackaged (B)		n = 45
	Retail outlet type	
Supermarket chain (SF)		n = 36
Fish market (FM)		n = 33
Specialised fish shop (SFS)		n = 30

^1^ number of samples.

**Table 2:** Prevalence of analysed bacteria in fish and seafood samples.

Bacteria	Fish n (%)	Seafood n (%)	Total n (%)
*Enterococcus* spp.	28 (48.3)	21 (51.2)	49 (49.5)
Presumptive VRE	8 (13.8)	9 (22)	17 (17.2)
*S. aureus*	43 (74.1)	37 (90.2)	80 (80.8)
Presumptive MRSA	13 (22.4)	14 (34.1)	27 (27.3)

(n) number of samples.

**Table 3:** Comparison of bacterial counts between fish and seafood samples (CFU/g).

Bacteria	Fish Median (Q1–Q3)	Seafood Median (Q1–Q3)	*p*-Value
*Enterococcus* spp.	<10 (<10–40)	<10 (<10–150)	0.219
Presumptive VRE	<10	<10	0.302
*S. aureus*	20 (2.5–25)	90 (20–210)	0.002
Presumptive MRSA	<10	<10 (<10–20)	0.220

**Table 4:** Influence of preservation status on bacterial counts (CFU/g).

Variable	Frozen Median (Q1–Q3)	Fresh Median (Q1–Q3)	*p*-Value
*Enterococcus* spp.	<10 (<10–40)	<10 (<10–100)	0.237
Presumptive VRE	<10	<10	0.215
*S. aureus*	30 (20–180)	30 (10–120)	0.216
Presumptive MRSA	<10 (<10–7.5)	<10	0.705

**Table 5:** Influence of packaging conditions on bacterial counts (CFU/g).

Variable	Packaged Median (Q1–Q3)	Unpackaged Median (Q1–Q3)	*p*-Value
*Enterococcus* spp.	<10 (<10–37.5)	30 (<10–110)	0.023
Presumptive VRE	<10	<10	0.194
*S. aureus*	20 (<10–142.5)	30 (20–180)	0.052
Presumptive MRSA	<10	<10	0.329

## Data Availability

The original contributions presented in this study are included in the article/Supplementary Materials. Further inquiries can be directed to the corresponding author.

## Supplementary Materials

The following supporting information can be downloaded at https://www.mdpi.com/article/10.3390/foods15142519/s1, Table S1. Microbiological results for individual fish and seafood samples analysed in the study.
